# Comparative study between PET-CT simulation and CT for head and neck cancer treatment and their potential effect on the thyroid gland: a prospective comparative study

**DOI:** 10.1186/s13014-026-02840-x

**Published:** 2026-05-18

**Authors:** Sameh Fawzy Nakhla, Youssef Abobker Abokasem, Hazem Elmansy, Tarek Mohamed Salem, Mohamed Fakhry Ahmed, Ebtsam Rizq Zaher, Mohamed Ibrahim Morsi

**Affiliations:** 1 Department of Radiation Sciences, Medical Research Institute, Alexandria University, Alexandria, Egypt; 2 Department of Radiology and Medical Imaging, Faculty of Allied Medical Science, Pharos University, Alexandria, Egypt; 3 Department of Cancer Management and Research, Medical Research Institute, Alexandria University, Alexandria, Egypt; 4 Department of Internal Medicine (Endocrinology Unit), Faculty of Medicine, Alexandria University, Alexandria, Egypt; 5 Department of Medical-Surgical Nursing, Faculty of Nursing, Alexandria University, Alexandria, Egypt

**Keywords:** Head and neck cancer, PET-CT simulation, Computed tomography (CT), Radiobiology, Radiotherapy, Thyroid gland, Radiation-induced thyroid dysfunction

## Abstract

**Background:**

Radiotherapy (RT) plays a key role in multidisciplinary treatment of Head and neck cancers (HNCs). Over the past two decades, advances have improved RT’s tumor control and toxicity profiles. Precise delineation of gross tumor volume and organs at risk using CT and PET-CT is crucial for safe and effective RT. Additionally, RT increases the risk of thyroid dysfunction, especially hypothyroidism.

**Aim:**

The present study compared PET-CT simulation and CT-alone planning in head and neck cancer (HNC) treatment, emphasizing their effects on thyroid gland function.

**Methods:**

The patients were divided into two groups according to the simulation method (PET-CT or CT), two fresh blood samples (5 mL/ sample) were collected from each patient, before and after radiotherapy. TSH, FT3, and FT4 (Thyroid biomarkers) were assessed by electrochemiluminescent immunoassay (ECLIA).

**Results:**

PET-CT simulation demonstrated a significant improvement in GTVp delineation accuracy by 71.8% compared with CT. After radiotherapy, TSH levels show minor decline changes, whereas FT3 and FT4 levels declined significantly, with greater declines observed in stages II and III. The most common tumor site was the larynx (60% in PET-CT, 75% in CT), followed by the tongue, nasopharynx, and parotid gland.

**Conclusion:**

PET-CT simulation offered superior GTVp delineation accuracy over CT, improving HNCs radiotherapy planning. Immediately after RT, radiotherapy caused significant declines in serum FT3 and FT4 levels, with only a minor, non-significant TSH declining, particularly in stage II/III disease. Notably FT4 is little more affected in PET-CT simulation patient group, highlighting the need for thyroid function monitoring. These results advocate PET-CT integration in planning and routine endocrine monitoring for treatment complications.

## Background

In recent years, Head and neck cancers (HNCs) have represented a significant global health issue with substantial incidence and mortality rates [1–4]. HNCs are among the most common malignancies globally, accounting for approximately (650,000- 660,000) new cases and (325,000- 330,000) deaths annually [3, 5].

Definitive radiotherapy (alone or concomitant radio chemotherapy) is commonly used as a cornerstone in the treatment of HNCs. For example, oropharyngeal, laryngeal, and hypopharyngeal squamous cell carcinomas (often within organ-preservation strategies), as well as nasopharyngeal carcinoma, where radiotherapy is the central curative modality. Radiotherapy is either used alone or concurrently with systemic treatment, mainly chemotherapy, or less frequently with targeted therapy [6–9]. New advancement in Radiotherapy techniques can effectively target tumors, thereby improving clinical outcomes and patient quality of life [10–12].

The introduction of Computed Tomography (CT) in the early 1970s represented a major breakthrough in medical imaging. It produces detailed cross-sectional images, enabling physicians to diagnose diseases with greater accuracy [13, 14]. Since the 1990s, CT has been utilized not only for diagnosis but also as the cornerstone of treatment planning in radiation therapy, providing the anatomical framework essential for target delineation and dose calculation [15, 16].

Magnetic resonance imaging (MRI) and CT are employed for standard examinations, staging, and follow-up in HNCs [17]. Both imaging techniques (CT and MRI) offer detailed anatomical information regarding the primary tumor and any metastases [17–20]. But recently, FDG-PET/CT has demonstrated greater accuracy compared to conventional methods [17, 21, 22]. The use of additional imaging techniques like MRI and PET enhanced the accuracy of target tissue identification, with some international guidelines established for this purpose [23, 24]. On the other hand, the combination of PET and CT technologies has significantly transformed medical imaging, especially in the management of cancer [14].

Multiple research centers have examined the role of 18F-FDG-PET in defining tumor target delineation for HNCs [25, 26]. Despite CT remains the gold standard for delineation of tumor volumes for radiation treatment planning [27], FDG-PET/CT combination has become an important tool in terms of target volume delineation [17, 28, 29].

In radiation therapy planning, tissues are generally classified into two main groups: those that require treatment and those that should be protected, known as organs at risk (OARs), one of them is the thyroid gland during radiotherapy treatment of HNCs (non-thyroidal) as radiation harms the thyroid by affecting its blood vessels, thyroid tissue cells, and triggering immune system responses [30, 31]. The ability to accurately minimize radiation exposure to OARs while maximizing the dose to the target tissues relies on how precisely the radiation oncologist delineates these tissues on each CT scan slice, as well as the dosimetrist’s expertise in creating a highly conformal treatment plan. This means that the prescribed dose closely matches the treatment volume, sparing surrounding healthy tissues as much as possible [24, 32].

The thyroid gland is particularly vulnerable to the damaging effects of radiation, which can result in a decrease in thyroid hormone production and the development of hypothyroidism [33]. Also, xerostomia is the most common side effect of radiotherapy for head and neck cancer, resulting from damage to the salivary glands. In addition, radiation-induced hypothyroidism represents one of the most clinically significant endocrine complications related to direct damage of thyroid tissue and its vasculature [30, 31, 34].

The thyroid function was evaluated by measuring thyroid hormones while the thyroid dose and volume are calculated by the radiotherapy planning system (RTPS). Dose–volume relationships are well described (e.g., mean thyroid dose, V30/V40/V50, etc.). A mean thyroid dose around ~ 30 Gy has been reported as a practical threshold and predictor of thyroid function changes [35].The use of PET-CT in RT planning in HNCs is very potential as its better identification of the disease extent (i.e. reduced geographical miss of radiation delivery to the gross tumor volume (GTV)). Also, characterizing the biological behavior of the disease [36].

To address this gap, the present study aimed to compare CT and PET-CT simulation techniques in the treatment planning of HNCs, with a particular focus on their potential impact on the radiation dose delivered to the thyroid gland. Specifically, the study looks to evaluate the differences in thyroid gland functions affection when using CT-based versus PET-CT-based simulation. Additionally, it aims to assess variations in organ delineation and GTV coverage between the two modalities.

## Subject and methods

Aim: The present study compares PET-CT simulation and CT-alone planning in head and neck cancer (HNC) treatment, emphasizing their effects on thyroid gland function. Study Type: A prospective, two-arm comparative clinical study. A priori power analysis using Epi Info (α =.05, power =.80, effect size d = 0.80) indicated a minimum required sample size of 24 patients per group, yielding a total of 48 patients. After applying inclusion and exclusion criteria, 49 patients were enrolled in the final analysis (PET-CT group: n = 25, CT group: n = 24). Patients were assigned to the PET-CT or CT simulation groups based on clinical indication and imaging availability. All patients in both groups met the following eligibility criteria: histologically confirmed non-thyroidal head and neck malignancy, normal baseline thyroid function tests (TSH, FT3, and FT4) prior to radiotherapy, and no prior history of thyroid disease or previous neck irradiation. Patients were randomly selected from the eligible patients those diagnosed (confirmed HNCs” nonthyroidal”) and/or treated at Alexandria University hospitals and NGO hospitals in Alexandria (from March 2023 to December 2024). Informed consent was obtained from all patients. The HNCs [49] patients were divided into two groups according to the simulation method; PET -CT (group (1): 25 HNCs (nonthyroidal) patients were simulated in treatment position on a PET-CT scanner [17] and CT simulation methods (group (2): 24 HNCs (nonthyroidal) patients that simulated using CT scanner [17, 25]. CT simulation was performed without contrast enhancement. Any patient with abnormal TSH, FT3, and/or FT4 level before Radiotherapy was excluded.

The CT dataset was imported into the treatment planning system for target and OAR delineation. Gross tumor volumes (GTV-primary and GTV-nodal), clinical target volumes (high-risk and elective nodal CTVs), and planning target volumes (PTVs) were defined using standard target-volume concepts and reporting principles. OARs were contoured on the planning CT, including the thyroid gland, and radiotherapy plans were generated using consistent prescription/optimization objectives; thyroid dose–volume histogram (DVH) metrics were exported for analysis. On the other hand, PET-guided cohort, 18 F-FDG PET/CT was incorporated into planning by registering a diagnostic PET-CT to the planning CT with registration quality verified prior to contouring. Metabolic information from PET was used to refine GTV delineation (particularly for nodal disease and equivocal primary extent), after which CTVs and PTVs were generated using the same expansion rules applied in the CT-only workflow. For PET-guided tumor delineation, A semi-quantitative fixed SUV threshold of 40% of SUVmax was used for PET-based tumor delineation. This threshold was applied consistently across all tumor sites to maintain standardization. However, manual adjustments were performed when necessary to account for physiological uptake and anatomical boundaries, considering the known spatial resolution limitations of PET imaging. Treatment plans were optimized to achieve comparable target coverage and OAR constraints across groups, and thyroid DVH metrics were recorded identically [17, 25, 37, 38].

Two fresh blood samples (5 mL/sample) were collected from each patient. The first sample was collected on the first day of radiotherapy prior to the initiation of the first treatment session, and the second sample was collected on the last day of radiotherapy immediately following the completion of the final treatment session. Venous blood was collected in a serum separating tube. The blood sample was allowed to clot for 30 min and then centrifuged at 3000 rpm for 10 min to isolate serum. The serum was stored in -20 °C freezer until got used to assay the serum biomarkers [39].

All the patients were with normal TSH, FT3, and FT4 prior to radiotherapy. Then, the circulating levels of thyroid-stimulating hormone [TSH] (REF:10995703) measured by chemilluninescent and detected by Atellica IM analyzer system according to (Simens healthineers Inc.) manufacturer’s instructions, Free triiodothyronine [FT3] (REF: 09005803190) by electrochemilluninescent immunoassay (ECLIA) and detected by Elecsys FT3 III system according to (Simens healthineers Inc.) manufacturer’s instructions, free thyroxine [FT4] (REF: 09043276190) by electrochemilluninescent immunoassay (ECLIA) and detected by Elecsys FT4 IV system according to (Simens healthineers Inc.) manufacturer’s instructions. Thyroid dose parameters such as Gy(mean)% per volume percentage of thyroid gland, v50 Gy %, v40 Gy %, GTVp were obtained from System Varian eclipse generated by the treatment planning system using IMRT technique.

All patients were treated using Intensity Modulated Radiation Therapy (IMRT) delivered via a Varian Unique linear accelerator (6 MV photon beams). The total prescribed dose ranged from 60 to 70 Gy, normalized to cover 95% of the Planning Target Volume (PTV). Conventional fractionation was applied at 2 Gy per fraction, with five fractions weekly, across a span of about 6 to 7 weeks. Hypofractionation used for selection of laryngeal cases was T1 larynx 2.25 Gy per fraction for 28 fractions, and T2 larynx received 2.25 Gy per fraction for 29 fractions. Treatment was delivered sequentially. The gross tumor volume (GTV) was expanded by 5–10 mm to create the clinical target volume (CTV), with an additional 3–5 mm margin to define the PTV. Elective nodal regions received 54–60 Gy. Tumor staging was performed according to the American Joint Committee on Cancer (AJCC) 8th edition TNM staging system.

### Statistical analysis

Data were fed into the computer and analyzed using IBM SPSS software package version 27.0. (Armonk, NY: IBM Corp, released in 2020). Categorical data were represented as numbers and percentages. Chi-square test was applied to compare between two groups. When more than 20% of the cells have expected to count less than 5, Fisher Exact correction test was applied for 2 × 2 table while the Monte Carlo correction was used for larger contingency tables. For continuous data, they were tested for normality by the Shapiro-Wilk test. Quantitative data were expressed as range (minimum and maximum), mean ± standard deviation (SD), Median and Inter quartile range (IQR) for normally distributed quantitative variables Student t-test was used to compare two groups while One way ANOVA test was used for comparing the more than two groups. Paired t-test was used to compare between two periods. On the other hand, for not normally distributed quantitative variables Mann Whitney test was used to compare two groups while Kruskal Wallis test was used to compare more than two groups. Significance of the results obtained was judged at the 5% level, and all tests were two-tailed.

## Result

### Demographic and clinicopathological patients’ characteristics

Tables 1 and 2 show that the two studied groups were of matched gender *p* = 0.725, age *p* = 0.765, family history (*p* = 0.171), stage (*p* = 0.208), and tumor type (*p* = 0.186). Table 3 illustrates the comparison of dose parameters between the two studied groups (total dose (*p* = 0.385), v50 Gy % (*p* = 0.575), v40 Gy % (*p* = 0.258)). Also, Table 4 demonstrated that the two studied groups were of matched hormonal levels: TSH (*p* = 0.937), FT3 (*p* = 0.273), and (FT4 *p* = 0.204).

**Table 1:** Comparison between the PET- CT (*n* = 25) and CT (*n* = 24) simulation according to demographic data

	PET- CT (*n* = 25)		CT (*n* = 24)		Test of Sig.	*p*
	No.	%	No.	%		
**Gender**						
Male	21	84.0	19	79.2	ꭓ^2^=0.191	^FE^*p*=0.725
Female	4	16.0	5	20.8		
**Age (years)**						
Min. – Max.	45.0–71.0		49.0–67.0		*t* = 0.300	0.765
Mean ± SD.	60.80 ± 6.52		60.33 ± 4.14			
Median (IQR)	61.0(57.0–65.0)		60.50(59.0–63.0)			
**Family history**						
No	22	88.0	17	70.8	ꭓ^2^=2.222	^FE^*p*=0.171
Yes	3	12.0	7	29.2		
**BMI (kg/m** ^**2**^ **)**						
Min. – Max.	21.30–31.60		18.30–35.40		*t* = 1.663	0.103
Mean ± SD.	26.30 ± 3.03		27.96 ± 3.91			
Median (IQR)	25.70(23.9–29.1)		28.25(25.6–30.7)			

IQR: Inter quartile range SD: Standard deviation *t*: Student *t*-test

χ^2^:Chi square test FE: Fisher Exact *p*: *p* value for comparing between the studied groups

**Table 2:** Comparison between PET- CT (*n* = 25) and CT (*n* = 24) simulation according to stage and diagnosis

	PET- CT (*n* = 25)		CT (*n* = 24)		ꭓ^2^	^MC^ *p*
	No.	%	No.	%		
**Clinical Stage**						
1	2	8.0	6	25.0	3.060	0.208
2	13	52.0	8	33.3		
3	10	40.0	10	41.7		
**Type of Cancer**						
Laryngeal	15	60.0	18	75.0	4.497	0.186
Nasopharyngeal	4	16.0	1	4.2		
Parotid	0	0.0	2	8.3		
Tongue	6	24.0	3	12.5		

χ^2^:Chi square test MC: Monte Carlo *p*: *p* value for comparing between the studied groups

**Table 3:** Comparison between PET- CT (*n* = 25) and CT (*n* = 24) simulation according to radiation dosimetry parameters

	PET- CT (*n* = 25)	CT (*n* = 24)	*U*	*p*
**Total dose (Gy)**				
Min. – Max.	60.0–70.0	50.0–70.0	260.50	0.385
Mean ± SD.	66.64 ± 3.80	67.13 ± 4.88		
Median (IQR)	66.0(65.0–70.0)	70.0(65.5–70.0)		
**Mean Dose (Gy)**				
Min. – Max.	12.0–69.0	6.0–72.0	256.50	0.384
Mean ± SD.	47.47 ± 14.94	50.43 ± 17.12		
Median (IQR)	52.0(38.0–57.2)	54.45(44.5–62.9)		
**v50Gy (%)**				
Min. – Max.	0.0–100.0	0.0–100.0	272.0	0.575
Mean ± SD.	57.48 ± 32.20	60.20 ± 37.03		
Median (IQR)	63.30(41.0–84.0)	78.40(29.0–90.9)		
**v40Gy (%)**				
Min. – Max.	13.0–100.0	1.0–100.0	244.0	0.258
Mean ± SD.	75.69 ± 26.91	77.58 ± 30.11		
Median (IQR)	91.0(54.0–97.0)	93.60(63.9–100.0)		

IQR: Inter quartile range SD: Standard deviation *U*: Mann Whitney test

*p*: *p* value for comparing between the studied groups *: Statistically significant at *p* ≤ 0.05

**Table 4:** Comparison between PET- CT (*n* = 25) and CT (*n* = 24) simulation for head and neck cancer according to thyroid function before and after radiation therapy

	Thyroid function	PET- CT (*n* = 25)	CT (*n* = 24)	*t*	*p*
**TSH (µIU/mL)**	**Before**				
	Min. – Max.	0.60–3.57	0.48–2.84	0.079	0.937
	Mean ± SD.	1.44 ± 0.70	1.45 ± 0.64		
	Median (IQR)	1.41(0.90–1.7)	1.37(0.90–1.9)		
	**After**				
	Min. – Max.	0.50–3.09	0.50–2.90	0.177	0.861
	Mean ± SD.	1.40 ± 0.62	1.44 ± 0.67		
	Median (IQR)	1.30(0.89–1.6)	1.36(0.85–1.8)		
	**p**_**1**_	0.337	0.702		
**Free T3 (pg/mL)**	**Before**				
	Min. – Max.	2.10–3.80	2.10–4.38	1.110	0.273
	Mean ± SD.	2.84 ± 0.48	3.01 ± 0.57		
	Median (IQR)	2.80(2.4–3.2)	2.93(2.7–3.5)		
	**After**				
	Min. – Max.	1.77–3.54	2.01–3.70	0.743	0.461
	Mean ± SD.	2.70 ± 0.45	2.80 ± 0.52		
	Median (IQR)	2.67(2.3–3.0)	2.74(2.4–3.3)		
	**p**_**1**_	< 0.001^*^	< 0.001^*^		
**Free T4 (ng/dL)**	**Before**				
	Min. – Max.	0.90–1.56	0.91–1.62	1.288	0.204
	Mean ± SD.	1.23 ± 0.19	1.30 ± 0.18		
	Median (IQR)	1.30(1.1–1.4)	1.36(1.2–1.4)		
	**After**				
	Min. – Max.	0.80–1.45	0.85–1.65	2.582^*^	0.013^*^
	Mean ± SD.	1.12 ± 0.17	1.25 ± 0.17		
	Median (IQR)	1.11(1.0–1.3)	1.24(1.2–1.3)		
	**p**_**1**_	< 0.001^*^	0.011^*^		

IQR: Inter quartile range SD: Standard deviation *t*: Student *t*-test *p*: *p* value for comparing between the studied groups

p_1_: *p* value for Paired *t*-test for comparing between Before and After in each group *: Statistically significant at *p* ≤ 0.05

### Thyroid function parameters

Table 4 illustrated that there is no significant difference in (TSH) levels between the PET and CT groups either before or after radiation therapy (*p* = 0.937 and *p* = 0.861, respectively). In contrast, free Triiodothyronine (free T3) levels significantly decreased in both groups (PET-CT and CT) after radiotherapy (*p* < 0.001), Similarly, free Thyroxine (free T4) levels declined significantly in both groups (PET-CT and CT) post-treatment (*p* < 0.001; *p* = 0.011 respectively). Notably, after RT, free T4 levels were significantly lower in the PET group 1.12 ± 0.17 compared to the CT group 1.25 ± 0.17 (*p* = 0.013). Overall, radiation therapy led to significant reductions in free T3 and free T4 levels in both groups (PET and CT) post radiotherapy, while TSH levels exhibited only a minor and statistically non-significant decline. While Table 5 Show no statistically significant difference between PET-CT and CT simulation from the reduction in thyroid function (TSH, Free T3, and Free T4) perspectives.

**Table 5:** Comparison between PET-CT and CT Simulation for Head and Neck Cancer according to thyroid function reduction before and after radiation therapy (*n* = 49)

Reduction in the Thyroid function	PET- CT (*n* = 25)	CT (*n* = 24)	Test of Sig.	*p*
**TSH (µIU/mL)**				
Min. – Max.	-0.20–0.48	-0.33–0.30	*t* = 0.373	0.711
Mean ± SD.	0.03 ± 0.16	0.01 ± 0.17		
Median (IQR)	0.04(-0.06–0.10)	0.02(-0.09–0.13)		
**Free T3 (pg/mL)**				
Min. – Max.	-0.15–0.50	-0.20–0.68	*t* = 1.248	0.218
Mean ± SD.	0.14 ± 0.18	0.21 ± 0.18		
Median (IQR)	0.10(0.03–0.26)	0.20(0.10–0.32)		
**Free T4 (ng/dL)**				
Min. – Max.	-0.08–0.41	-0.19–0.24	*U* = 248.50	0.302
Mean ± SD.	0.11 ± 0.13	0.05 ± 0.09		
Median (IQR)	0.07(0.02–0.10)	0.06(0.02–0.10)		

IQR: Interquartile range SD: Standard deviation *t*: Student *t*-test *p*: *p*-value for comparing between the studied groups

*U*: Mann Whitney test

Table 6 Show no significant changes in TSH levels, and the differences between groups were not statistically significant. However, significant reductions in free T3 and T4 levels were noted in stages II and III. These findings suggest that radiotherapy had a notable impact on Free T3 and Free T4 levels, particularly in more advanced stages of cancer while TSH Show minor decline changes. The study revealed that while TSH levels remained stable post-radiotherapy (*p* > 0.05), there was a significant reduction in Free T3 (*p* < 0.001) and Free T4 (*p* = 0.002) for the laryngeal group and a significant decline in Free T4 (*p* = 0.034) for the tongue group. Since no significant differences were found between the two cancer sites (*p* > 0.05) as illustrated in Tables 7 and 8.

**Table 6:** Comparison of thyroid function parameters according to patient clinical stages

	Thyroid function	Clinical stage			*F*	*p*
		I (*n* = 8)	II (*n* = 21)	III (*n* = 20)		
**TSH (µIU/mL)**	**Before**					
	Min. – Max.	0.94–3.57	0.48–2.84	0.60–2.50	0.936	0.400
	Mean ± SD.	1.67 ± 0.84	1.31 ± 0.67	1.49 ± 0.58		
	Median	1.39	1.12	1.45		
	**After**					
	Min. – Max.	0.90–3.09	0.50–2.90	0.50–2.45	0.643	0.530
	Mean ± SD.	1.60 ± 0.73	1.31 ± 0.69	1.46 ± 0.55		
	Median	1.36	0.98	1.46		
	**p**_**1**_	0.387	0.931	0.400		
**Free T3 (pg/mL)**	**Before**					
	Min. – Max.	2.32–3.50	2.10–4.38	2.10–3.70	1.509	0.232
	Mean ± SD.	2.94 ± 0.37	3.06 ± 0.59	2.78 ± 0.50		
	Median	2.95	3.0	2.79		
	**After**					
	Min. – Max.	2.33–3.30	1.77–3.70	2.01–3.50	1.009	0.372
	Mean ± SD.	2.81 ± 0.38	2.84 ± 0.54	2.63 ± 0.47		
	Median	2.90	2.67	2.60		
	**p**_**1**_	0.130	< 0.001^*^	< 0.001^*^		
**Free T4 (ng/dL)**	**Before**					
	Min. – Max.	0.91–1.53	0.90–1.62	0.96–1.47	0.421	0.659
	Mean ± SD.	1.21 ± 0.22	1.27 ± 0.21	1.28 ± 0.14		
	Median	1.23	1.32	1.30		
	**After**					
	Min. – Max.	0.85–1.45	0.80–1.65	0.89–1.40	0.020	0.980
	Mean ± SD.	1.17 ± 0.20	1.19 ± 0.21	1.19 ± 0.14		
	Median	1.21	1.15	1.22		
	**p**_**1**_	0.155	0.002^*^	0.005^*^		

IQR: Inter quartile range SD: Standard deviation *F*: *F* for One way ANOVA test *p*: *p* value for comparison between the categories studied

p_1_: *p* value for Paired *t*-test for comparing between Before and After in each category

**Table 7:** Thyroid function results (TSH, Free T3, and Free T4) before and after RT across four studied subgroups (Tumor type): laryngeal, nasopharyngeal, parotid, and tongue

	Thyroid function	Tumor type			
		Laryngeal (*n* = 33)	Nasopharyngeal (*n* = 5)	Parotid (*n* = 2)	Tongue (*n* = 9)
**TSH (µIU/mL)**	**Before**				
	Min. – Max.	0.61–3.57	0.60–2.30	0.99–2.84	0.48–2.40
	Mean ± SD.	1.46 ± 0.64	1.42 ± 0.63	1.92 ± 1.31	1.30 ± 0.72
	Median	1.38	1.41	1.92	0.90
	**After**				
	Min. – Max.	0.50–3.09	0.50–2.24	1.06–2.90	0.70–2.20
	Mean ± SD.	1.44 ± 0.62	1.31 ± 0.64	1.98 ± 1.30	1.30 ± 0.63
	Median	1.30	1.38	1.98	0.89
**Free T3 (pg/mL)**	**Before**				
	Min. – Max.	2.18–3.80	2.10–3.86	3.20–3.70	2.10–4.38
	Mean ± SD.	2.90 ± 0.44	3.06 ± 0.81	3.45 ± 0.35	2.83 ± 0.67
	Median	2.88	3.44	3.45	2.77
	**After**				
	Min. – Max.	2.01–3.54	1.77–3.66	3.02–3.56	2.10–3.70
	Mean ± SD.	2.74 ± 0.42	2.86 ± 0.84	3.29 ± 0.38	2.62 ± 0.49
	Median	2.69	3.18	3.29	2.59
**Free T4 (ng/dL)**	**Before**				
	Min. – Max.	0.91–1.56	0.90–1.40	1.25–1.62	1.11–1.62
	Mean ± SD.	1.24 ± 0.18	1.27 ± 0.22	1.44 ± 0.26	1.32 ± 0.15
	Median	1.27	1.40	1.44	1.30
	**After**				
	Min. – Max.	0.85–1.45	0.80–1.40	1.13–1.54	0.90–1.65
	Mean ± SD.	1.17 ± 0.16	1.19 ± 0.24	1.34 ± 0.29	1.18 ± 0.21
	Median	1.21	1.30	1.34	1.12

**Table 8:** Thyroid function results (TSH, Free T3, and Free T4) before and after RT across two of the studied subgroups (Tumor type): laryngeal, and tongue

	Thyroid function	Diagnosis		*t*	*p﻿*
		Laryngeal (*n* = 33)	Tongue (*n* = 9)		
**TSH**	**Before**				
	Min. –Max.	0.61–3.57	0.48–2.40	0.648	0.521
	Mean ± SD.	1.46 ± 0.64	1.30 ± 0.72		
	Median	1.38	0.90		
	**After**				
	Min. –Max.	0.50–3.09	0.70–2.20	0.578	0.567
	Mean ± SD.	1.44 ± 0.62	1.30 ± 0.63		
	Median	1.30	0.89		
	**p**_**1**_	0.481	0.938		
**Free T3**	**Before**				
	Min. –Max.	2.18–3.80	2.10–4.38	0.370	0.713
	Mean ± SD.	2.90 ± 0.44	2.83 ± 0.67		
	Median	2.88	2.77		
	**After**				
	Min. –Max.	2.01–3.54	2.10–3.70	0.686	0.497
	Mean ± SD.	2.74 ± 0.42	2.62 ± 0.49		
	Median	2.69	2.59		
	**p**_**1**_	< 0.001^*^	0.052		
**Free T4**	**Before**				
	Min. –Max.	0.91–1.56	1.11–1.62	1.304	0.200
	Mean ± SD.	1.24 ± 0.18	1.32 ± 0.15		
	Median	1.27	1.30		
	**After**				
	Min. –Max.	0.85–1.45	0.90–1.65	0.156	0.877
	Mean ± SD.	1.17 ± 0.16	1.18 ± 0.21		
	Median	1.21	1.12		
	**p**_**1**_	0.002^*^	0.034^*^		

SD: Standard deviation *t*: Student *t*-test

*p*: *p*-value for comparison between the studied categories

*p*_1_: *p*-value for the Paired *t*-test for comparing between Before and After in each category

*: Statistically significant at *p* ≤ 0.05

### Thyroid radiation does parameters

**Fig. 1:**
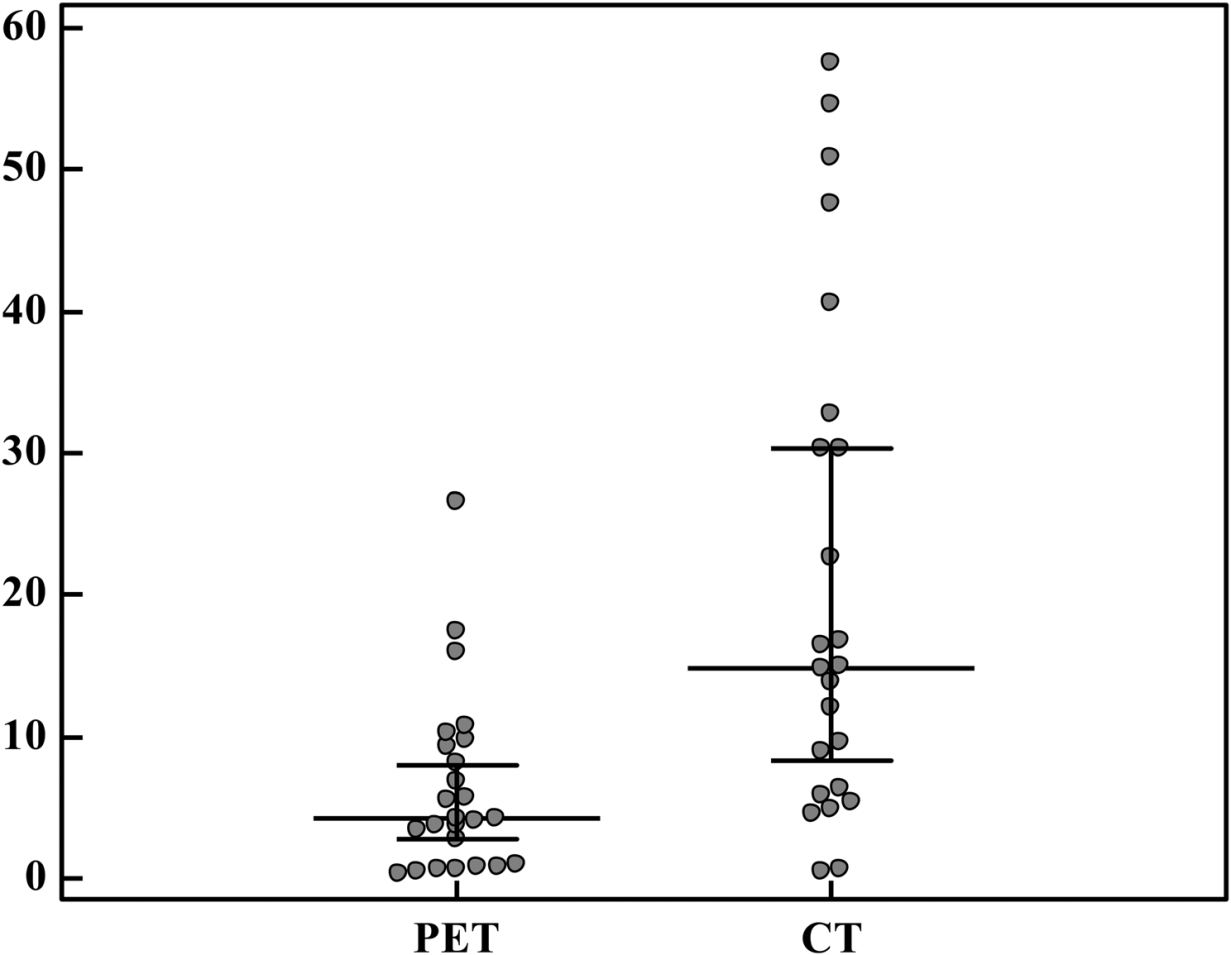
Comparison between the two studied groups according to GTV

Table 9 and Fig. 1 illustrated a significant difference in gross tumor volume (GTVp) between the PET and CT groups (*p* = 0.001). The GTVp measured by PET was considerably smaller, with a mean of 6.30 ± 6.33, compared to 20.95 ± 17.85 in the CT group. These results demonstrated that tumor volumes identified on PET scans are significantly smaller than those identified by CT scans and PET-CT simulation improved the GTVp by 71.8% i.e. from 14.9(6.1–31.6) to 4.2(0.9–9.3).

**Table 9:** Comparison between PET- CT (*n* = 25) and CT (*n* = 24) Simulation according to gross tumor volume (GTVp)

	PET- CT (*n* = 25)	CT (*n* = 24)	*U*	*p﻿*
**GTVp**				
Min. – Max.	0.40–26.60	0.50–57.50	126.0	0.001^*^
Mean ± SD.	6.30 ± 6.33	20.95 ± 17.85		
Median (IQR)	4.20(0.90–9.3)	14.90(6.1–31.6)		

IQR: Inter quartile range SD: Standard deviation *U*: Mann Whitney test

*p*: *p* value for comparing between the studied groups *: Statistically significant at *p* ≤ 0.05

Table 10 Demonstrated the relation between radiation dose parameters and four different tumor sites: laryngeal, nasopharyngeal, parotid, and tongue. Statistically significant differences were observed in both the mean dose (Gy) and the volume of tissue receiving at least 50 Gy (v50Gy) (*p* = 0.001 and 0.002 respectively). Patients with laryngeal and nasopharyngeal cancers received higher mean doses and had a larger percentage of tissue exposed to 50 Gy compared to those with parotid and tongue cancers.

**Table 10:** Relation between tumor type and the thyroid different radiation dose parameters (*n* = 49)

	Tumor type				H	*p*
	Laryngeal (*n* = 33)	Nasopharyngeal (*n* = 5)	Parotid (*n* = 2)	Tongue (*n* = 9)		
**Mean Dose (Gy)**						
Min. – Max.	12.0–72.0	40.50–57.20	6.0–9.0	26.0 − 54.10	15.839^*^	0.001^*^
Mean ± SD.	53.62 ± 14.17	51.46 ± 6.80	7.50 ± 2.12	39.48 ± 8.67		
Median	56.0	52.0	7.50	39.0		
**v50Gy (%)**						
Min. – Max.	5.50–100.0	0.0 − 93.40	0.0–3.50	0.0–80.80	15.107^*^	0.002^*^
Mean ± SD.	70.45 ± 28.51	60.94 ± 36.45	1.75 ± 2.47	27.64 ± 26.03		
Median	82.80	64.0	1.75	28.0		
**v40Gy (%)**						
Min. – Max.	11.0–100.0	62.80– 98.0	1.0–86.0	34.0 − 99.40	5.446	0.142
Mean ± SD.	79.75 ± 28.52	86.0 ± 14.87	43.50 ± 60.10	67.27 ± 22.36		
Median	95.0	91.0	43.50	68.0		

IQR: Inter quartile range SD: Standard deviation H: H for Kruskal Wallis test *p*: *p* value for comparison between the studied categories

## Discussion

About 2.68% of all cancer cases and 2.22% of all cancer-related fatalities in Egypt were caused by HNCs, primarily squamous cell cancer (Ibrahim & Shash, 2022; Nawar et al., 2024). Head and neck anatomy is complex and includes many OARs. To treat patients optimally, target volumes must be delineated precisely in order to deliver high radiation dose to macroscopic disease while limiting excessive dose to the OARs. This increasing precision requires evaluation of target-volume delineation, which is currently based on physical examination, computed tomography, and magnetic resonance imaging. The introduction of 18 F-fluorodeoxyglucose (18 F-FDG) positron emission tomography (PET) in the last two decades has increased the accuracy in cancer management [25]. 18FDG-PET has been increasingly used in radiation oncology since integration of 18FDG-PET and CT imaging, which allows for simultaneous utilization of metabolic and anatomic data [40]. PET images are characterized by high contrast but low spatial resolution, which creates a need for combining PET with morphological imaging methods such as cone beam computed tomography (CBCT) [40]. Consequently, the use of PET imaging in radiotherapy treatment might change the dosimetric tumor coverage with respect to a conventional CT-based treatment plan [17]. How different the dosimetric tumor coverage in a PET-CT-based treatment plan is compared to a CT-based treatment plan is the overall question that we attempted to answer in the present study as applied to HNCs.

Hussein et al., 2018 [41] who aimed to compare between 18F-FDG-PET/CT versus CT scan in GTV volumes delineation in patients with HNC. The study included 30 patients with disease stages I, II, III, and IVa. Each patient underwent a combined 18F-FDG-PET/CT scan while immobilized in the treatment position to simulate radiotherapy setup. Results Show that the most common tumor site was nasopharynx (36.6%), followed by oropharynx, oral cavity, larynx, hypopharynx, and cervical lymphadenopathy of unknown primary. In addition to the median primary tumor, GTV delineated on CT (CT-GTVp) was larger than that on PET-CT (PET-GTVp) 32.1 cc versus 18.5 cc with a statistically significant difference (*p* = 0.0026). This corresponds to about a 30.08% reduction in tumor volume when using PET-CT to delineate the tumor. i.e. PET-CT imaging tends to produce smaller gross tumor volumes for the primary tumor compared to CT alone, which may allow for more precise targeting in radiotherapy and potentially better sparing of normal tissues. Hussein et al., 2018 [41] results were parallel with ours. The present study observed that the GTVp measured by PET-CT was considerably smaller, with a mean of 6.30 ± 6.33, compared to 20.95 ± 17.85 in the CT group. These results demonstrated that tumor volumes identified on PET-CT scans were significantly smaller than those identified by CT scans and PET-CT simulation improved the GTVp by 71.8%. In The present study the most common tumor site was Laryngeal (60%, 75%) in PET-CT and CT respectively, followed by Tongue, Nasopharyngeal and Parotid. In conclusion, both studies Hussein et al., 2018 [41] and The present study confirmed that the combined PET-CT improves the accuracy of primary tumor volume delineation compared to CT alone in HNCs radiotherapy planning, possibly leading to better treatment outcomes by reducing unnecessary radiation to surrounding healthy tissues [41].

Bernat & Hrusak, 2014 [42] study aimed to evaluate the frequency of thyroid dysfunction in patients with HNCs who underwent radiotherapy, and to assess the temporal development of thyroid hypofunction following radiation. Their study included: 43 patients who underwent radiotherapy with or without surgery. These patients had more advanced stages (III and IV) or recurrent cancer with histologically confirmed non-thyroid head and neck malignancies. Results demonstrated a substantial incidence of hypothyroidism at 35% among irradiated patients, confirming that radiotherapy to the head and neck is a significant risk factor for thyroid dysfunction. Among these (35%), 40% had subclinical hypothyroidism and 60% had clinical hypothyroidism and (7%) in the RT group had euthyroid sick syndrome. In The present study, patient groups were carefully matched according to gender, age, family history, tumor stage, and type, as well as baseline thyroid hormone levels (TSH, FT3, and FT4). Similar to Bernát and Hrušák’s findings, no significant differences related to gender or age were observed in thyroid function changes post-radiotherapy, reinforcing these factors as non-predictive for hypothyroidism development. Notably, Bernat & Hrusak, 2014 [42] retrospective analysis reported a significant increase in TSH levels over time along with declines in FT4 (and a less consistent decline in FT3), The present study results Show stable TSH levels after radiotherapy but significant reductions in both FT3 and FT4 particularly pronounced in patients with stage II and III disease compared to stage I, suggesting that more advanced tumor stage may influence the degree of thyroid hormone impairment. Additionally, in patients with tongue tumors, only FT4 exhibited significant change post-treatment, whereas both FT3 and FT4 were affected overall; this partial site-specific variation was not explored in Bernat & Hrusak, 2014 study. These observations collectively suggest that while radiotherapy induced thyroid dysfunction is a well-established late effect, the dynamics of hormone changes can vary depending on disease stage and tumor location, emphasizing the need for individualized monitoring strategies in HNC patients receiving radiation [42].

The acute decline in serum FT3 and FT4 levels observed immediately following radiotherapy in the present study presents an interesting contrast to some previous reports. For instance, Bakhshandeh et al. (2012) observed a transient increase in FT4 levels toward the end of a 6–7 week radiotherapy course, attributed to the release of stored hormones [43]. The immediate post-RT decline found in our study may not represent primary hypothyroidism, but rather an acute phase response. This highlights that thyroid hormone alterations in HNC patients may follow a more complex temporal pattern than previously described, necessitating both immediate and long-term surveillance.

Both (The present study and Nawar et al., 2024) addressed the impact of radiotherapy on thyroid function in HNC patients. The present study Show stable TSH levels post-radiotherapy, whereas FT3 and FT4 declined significantly, especially in stage II and III tumors, with FT4 changes noted in tongue tumors and both FT3 and FT4 significantly affected in laryngeal and nasopharyngeal cancers. Nawar et al., 2024 similarly demonstrated progressive thyroid dysfunction after radiotherapy, with TSH rising significantly (*p* < 0.001*) at 6 and 12 months along (3.05 ± 1.67, 5.67 ± 4.39 respectively) with FT4 decline, confirming a high risk of hypothyroidism that increases over time; subclinical hypothyroidism was common at 6 months and progressed to clinical hypothyroidism by 12 months in nearly a quarter of patients. In The present study, we highlighted that PET-CT simulation improving gross tumor volume delineation, thus potentially enhancing radiotherapy precision, Nawar et al., 2024 focused on conventional CT-based 3D conformal radiotherapy. Both studies emphasize the critical role of thyroid dosimetry parameters as predictors of radiation-induced hypothyroidism. Also, the main difference was that Nawar et al., 2024 measured the thyroid function biomarkers at 6- and 12-months postradiotherapy but ours was prospective study and the changes were assessed immediately after RT. In addition to that we underscored that thyroid function deteriorates progressively after radiotherapy, highlighting the need for routine thyroid monitoring and the importance of dosimetric planning to minimize OAR exposure and mitigate long-term complications [30].

Delouya et al., 2011 aimed to evaluate the impact of incorporating 18F-FDG-PET/CT into radiotherapy planning for head and neck squamous cell carcinoma, focusing on whether PET-CT-based contouring altered gross tumor volumes and potentially changed patient management. In their prospective cohort study of 29 patients, PET-CT significantly reduced the mean primary gross tumor volume (GTVp) compared with CT alone (18 cc “18F-FDG-PET” vs. 24 cc “CT”; (*p* = 0.001)), with a decrease observed in 80% of cases (GTVp-PET was smaller than the GTVp-CT in 80% of the cases). These findings aligned with our results which demonstrated that PET-CT simulation yielded a markedly larger median reduction in GTVp (71.8%, from 14.90 [6.1–31.6] to 4.20 [0.90–9.3]) compared with CT simulation. But The present study was designed to assess not only the effect of simulation modality (PET-CT vs. CT) on tumor volume delineation but also to explore its relationship with thyroid radiation dose parameters and subsequent thyroid function changes [25].

The aim of the study conducted by Samołyk-Kogaczewska et al., 2019 was to evaluate the usefulness and accuracy of hybrid 18 F-FDG PET/MRI in gross tumor volume (GTV) delineation during radiotherapy planning in patients with squamous cell carcinoma (SCC) of the tongue. Results demonstrated that PET/MRI provided more detailed and accurate tumor volume delineation compared to CT and MRI alone, with the 30% SUVmax threshold being most frequently aligned with CT-based GTV for the primary tumor, and 30–40% thresholds optimal for nodal volumes. Their findings revealed that PET/MRI often identified tumor infiltrations not visible on other imaging modalities, thus improving targeting precision. In line with this, these study results Show that PET-CT simulation improved the primary GTV by 71.8%, reducing the volume from a median of 14.90 to 4.20, indicating enhanced delineation accuracy [40].

Delouya et al., 2011, Venkada et al., 2012, Ferrando et al., 2018 and Hussein et al., 2018 demonstrated that the combined PET-CT improves the accuracy of primary tumor volume (GTVp) delineation compared to CT alone in HNCs radiotherapy planning, possibly leading to better treatment outcomes by reducing unnecessary radiation to surrounding healthy tissues [17, 25, 41, 44]. Beyond primary tumor delineation, PET/CT allowed for more precise identification of both the primary tumor and metastatic lymph nodes. In addition to improving delineation of the primary lesion through detection of metabolically active tumor tissue, PET/CT also identified lymph node metastases that were not clearly visible on CT alone, particularly in cases where nodes were not enlarged but demonstrated increased FDG uptake. This resulted in more accurate staging and target volume definition. However, despite improved detection of nodal disease, this did not translate into a significant difference in radiation dose delivered to the thyroid gland between the PET and CT groups.

Most of the previously mentioned studies didn’t assess the thyroid biomarkers immediately after RT, but Nakhla et al.2022, results Show significant alterations in thyroid function parameters correlated with radiation exposure. Specifically, TSH and IGF-1 levels significantly decreased during and after radiotherapy compared to controls, while FT4 levels increased significantly across the treatment timeline. On the other hand, FT3 did not differ significantly between patients and controls, suggesting that radiation primarily influenced TSH and FT4 without invoking the typical feedback mechanism. Despite Nakhla et al.2022 worked on children samples, both studies (The present study and Nakhla et al.2022) were consistent that both are prospective studies and levels of thyroid gland hormones were measured immediately after RT. The present study found that TSH levels remained stable after radiotherapy but significant reductions in both FT3 and FT4 particularly pronounced in patients with stage II and III disease compared to stage I. Additionally, in patients with tongue tumors, only FT4 exhibited significant change post-treatment, whereas both FT3 and FT4 were affected overall [45].

Beyond thyroid dysfunction, Incidental irradiation of the hypothalamic–pituitary axis may lead to hypopituitarism and deficiencies in multiple hormonal pathways (Hilal et al., 2021). Radiation-induced adrenal insufficiency has also been reported following stereotactic ablative radiotherapy of adrenal metastases [46]. Furthermore, pelvic radiotherapy has been associated with premature ovarian insufficiency in young women and alterations in male sex hormone levels [47, 48]. Even abdominal radiotherapy has been shown to deliver significant incidental doses to the pancreas, with potential implications for endocrine pancreatic function [49]. These findings collectively emphasize the need for comprehensive endocrine evaluation and long-term follow-up in all patients receiving radiotherapy, regardless of the primary tumor sit.

## Conclusion

This comparative study found that PET-CT simulation significantly improved the accuracy of (GTVp) delineation by 71.8% compared to CT, indicating its value in optimizing HNCs treatment planning. While the observed GTVp volume reduction does not inherently prove superior accuracy, the integration of PET-CT provides essential metabolic data that refines target definition. Thyroid function analysis Show that thyroid function levels declined significantly after radiotherapy (minor decline TSH, and declined FT3& FT4, these results could be due to secondary hypothyroidism (Pituitary in origin) or sick euthyroid syndrome), with greater alterations observed in stage II and III disease compared to stage I. Laryngeal cancer was the most common tumor site in both modalities, highlighting the clinical advantage of PET-CT in precise tumor targeting and the need for careful thyroid function monitoring in HNC patients.

### Limitations

Study limitations include the small size of certain tumor subgroups (e.g., nasopharyngeal, *n* = 5; parotid, *n* = 2), which are presented as preliminary descriptive data rather than definitive conclusions. Furthermore, thyroid function monitoring in the present study was limited to the immediate post-radiotherapy period which focused on the immediate post-radiotherapy period according to the study’s initial scope. Additionally, the nodal involvement was determined based on combined PET/CT metabolic and morphological criteria according to standard institutional practice.

## Data Availability

The datasets and/or analyses of the current study are available from the corresponding author on reasonable request.

## Abbreviations

BMI: Body Mass Index
CBCT: Cone Beam Computed Tomography
CT: Computed Tomography
ECLIA: Electrochemiluminescent Immunoassay
FDG: Fluorodeoxyglucose
FT3: Free Triiodothyronine
FT4: Free Thyroxine
GTV: Gross Tumor Volume
GTVp: Gross Tumor Volume (primary)
HNCs: Head and Neck Cancers
MRI: Magnetic Resonance Imaging
OAR: Organs at Risk
PET-CT: Positron Emission Tomography–Computed Tomography
RT: Radiotherapy
TSH: Thyroid Stimulating Hormone
